# Unicystic Ameloblastoma (UA): A Case Series

**DOI:** 10.7759/cureus.31039

**Published:** 2022-11-03

**Authors:** Padmashri P Kalmegh, Alka H Hande, Madhuri N Gawande, Swati K Patil, Archana M Sonone

**Affiliations:** 1 Oral and Maxillofacial Pathology and Microbiology, Sharad Pawar Dental College and Hospital, Datta Meghe Institute of Medical Sciences, Wardha, IND

**Keywords:** intraluminal proliferation, odontogenic origin, multilocular radiolucency, luminal proliferation, unicystic ameloblastoma

## Abstract

Ameloblastomas are true benign tumors of odontogenic epithelial origin mostly seen in the mandible. After odontoma, it is the second most commonly seen odontogenic neoplasm. Ameloblastomas comprise several clinical, radiological, and histological varieties, making them the most significant odontogenic neoplasm. Unicystic ameloblastomas (UAs) refer to those cystic lesions that show clinical, radiographic, or gross features of jaw cysts but on histologic examination, they show a typical ameloblastomatous epithelium lining the cysts' cavities, with or without luminal and/or mural tumor proliferation. UAs are a less encountered variant of ameloblastomas and are believed to be less aggressive. As this tumor shows considerable similarities with dentigerous cysts, both clinically and radiographically the biological behavior of this tumor group was reviewed.

## Introduction

Ameloblastomas are true benign neoplasm that originates from odontogenic epithelium. An ameloblastoma is likely to progress to a large size, with resultant bone deformity or bone defect. It is categorized into unicystic, multicystic, peripheral, and malignant ameloblastomas [1]. One of the most important subtypes is unicystic ameloblastomas (UAs). The prevalence of UAs is in the posterior mandibular region as around 90% of cases occur in the mandible. About 50-80% of UAs are associated with an impacted tooth [2]. Considering the background of diversity in histological presentation and biological behavior, the prognosis of these tumors is uncertain. UAs respond favorably to conservative treatment, which includes enucleation and marsupialization in certain extensive lesions [3]. Here we present an unusual case series of UAs having the varied type of growth in the mandible with added emphasis on their histopathological importance.

## Case presentation

Case 1

A 34-year male presented with a complaint of swelling over the left mandibular region for seven-to-eight months. He was alright seven-to-eight months ago but later observed a swelling with a current size of grossly 2 x 3 cm. He first visited a private hospital in Akola, India, where a cone beam computed tomography (CBCT) scan suggested ameloblastoma. The patient did not give a history of trauma, difficulty in mastication, or change in consistency and quantity of saliva.

**Figure 1:**
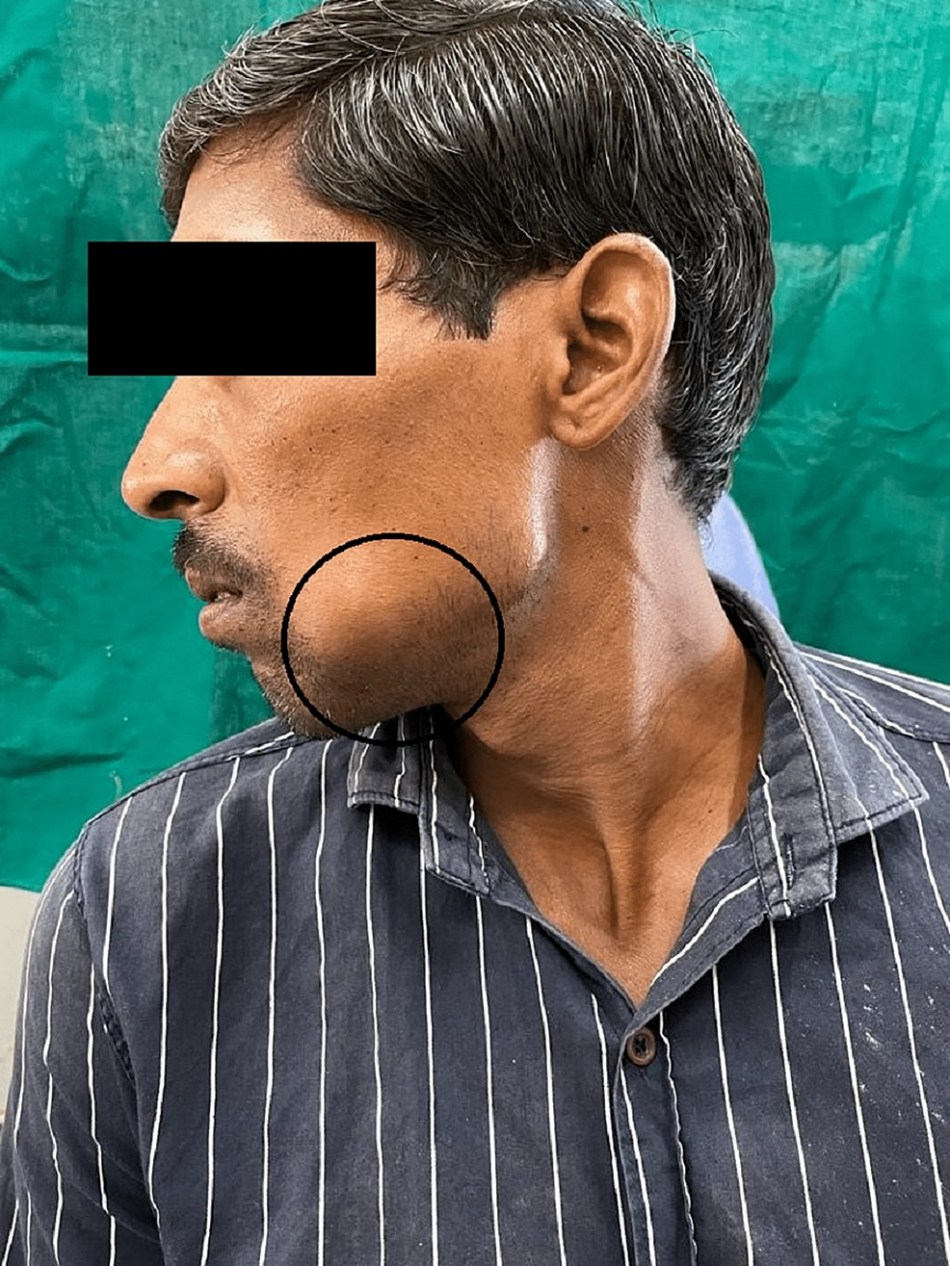
Oval swelling with diffuse border

Extraorally, approximately oval, diffuse, hard, and non-tender swelling was palpated over the left corner of the mouth. Anteroposteriorly, swelling extended behind the left corner of the mouth to 3 cm short of the angle of the mandible. Superoinferiorly, swelling extended below the upper lip to 1 cm short of the inferior border of the mandible (Figure 1). The temporomandibular joint (TMJ) was bilaterally synchronous with no clicking sound or deviation. Left submandibular lymph nodes were palpable with the size of 1 x 2 cm, non-tender, and hard in consistency. The overlying skin was not involved by the swelling and no rise in local temperature was associated with it. Intraorally, a well-defined, non-mobile, non-tender, firm-to-hard swelling was present. 

Radiographic Features

To diagnose ameloblastoma and to determine its extension, radiographic interpretation is considered an important factor.

**Figure 2:**
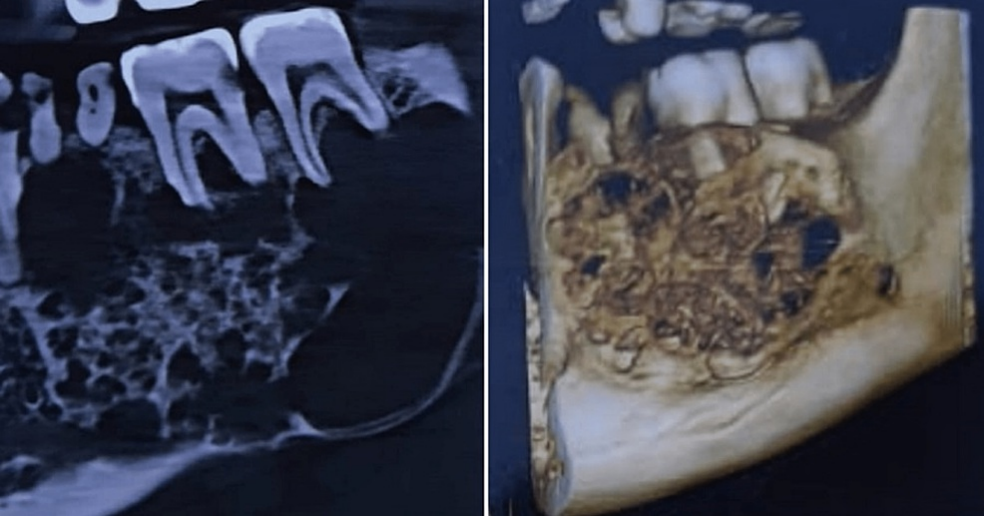
Multilocular radiolucency with honeycomb appearance

A single quadrant CBCT presented a multilocular radiolucency with well-defined borders, having a honeycomb appearance over the left body of the mandible and extending anterioposteriorly from the distal surface of 35 to the distal surface of the 38 and superiorly from apices of root stump of 36 to the inferior border, not surrounded by any sclerotic border. Trabeculae were present within lesion. Inferior extension thinned out the border of the mandible and resulted in root resorption of 38 and displacement of the inferior alveolar nerve (Figure 2).

Histopathologic Features

**Figure 3:**
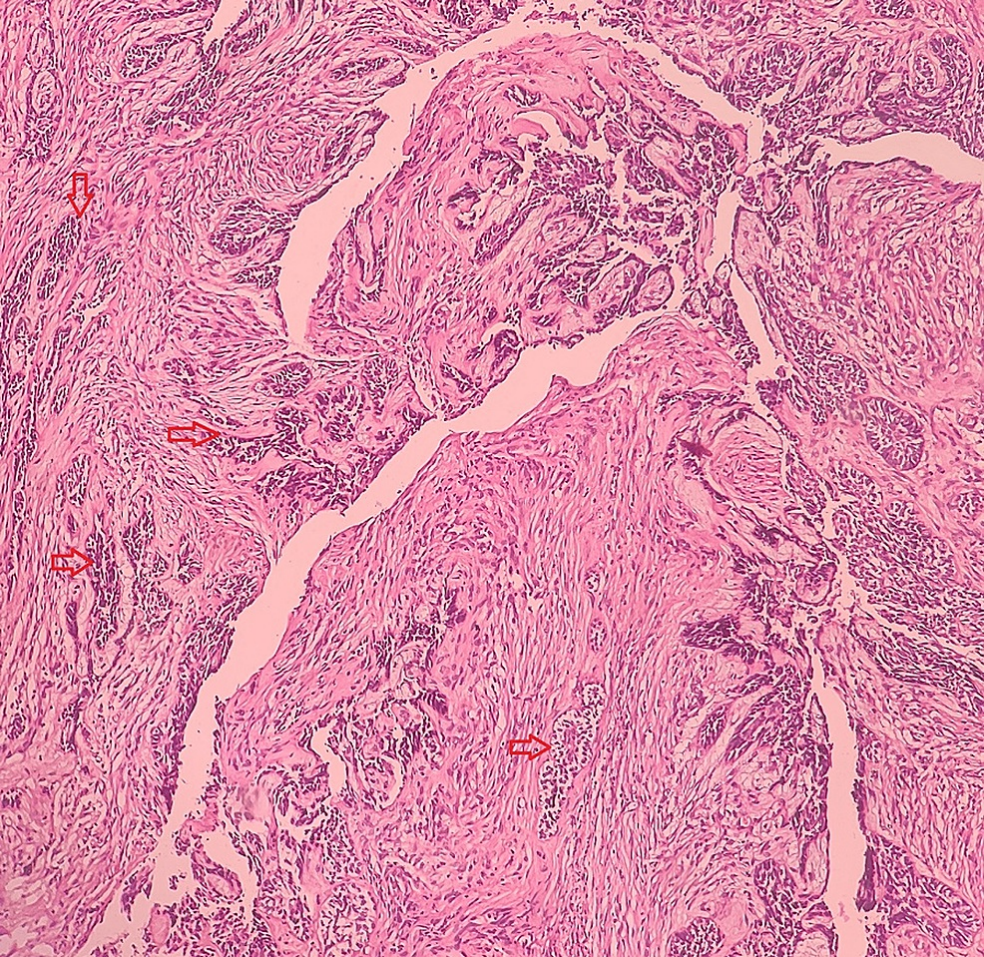
Intramural variation

UAs are categorized into several histologic subtypes depending on the pattern and proliferation of neoplastic cells within the cyst wall. Three histological variants of UAs are: the luminal variant, if the extension of tumor is limited to the luminal surface of the cyst or the cystic cavity is lined by ameloblastic epithelium; intraluminal variant, if the ameloblastomatous nodules protrude into the cystic lumen; and mural variant, if the tumor is infiltred into the cystic wall. On histopathological examination, Case 1 shows luminal and intramural types of ameloblastomatous proliferation. In the present case, the luminal areas of the tumor are lined by tall columnar ameloblast like cells with hyperchromatic nuclei and prominent subnuclear vacuolization. The intramural area shows infiltration of the cyst lining by tumor cells with central cystic degeneration suggestive of UAs, Subtype 1.3 (Figure 3).

Treatment

The recurrence rate for UAs is highly variable. As intramural subtype cannot be identified preoperatively, more aggressive treatment is recommended. For treatment modality, considering the large and expansive nature of this lesion a radical section was a choice of treatment. Reconstruction of the defect with fibular graft was done. As patient was managed by radical section, to maintain the occulsal stability and to prevent the pathological fracture, reconstruction was done with titanium plates. During follow up, the patient had presented with trismus, which was treated with myofunctional therapy with good evaluation and functionality of the jaw, without esthetic sequelae.

Case 2

A 32-year-old man came to our institute with swelling and associated pain over the right angle of the mandible for the past one year. Pain was intermittent, dull, chronic, and radiating in nature. Extraorally, a solitary diffuse swelling of size 4 X 3.5 cm palpated over the right angle of the mandible. Anteroposteriorly, it extended behind the right corner of the mouth to the ramus of mandible. Superoinferiorly it was below the canthus of the eye up to the inferior border of the mandible. The overlying skin was free of any inflammatory sign. On intraoral examination, bony, hard, and tender swelling was evident over the right alveolus involving a distal surface of 44 to a distal surface of 48 obliterating the right buccal and lingual vestibule.

Radiographic Features

**Figure 4:**
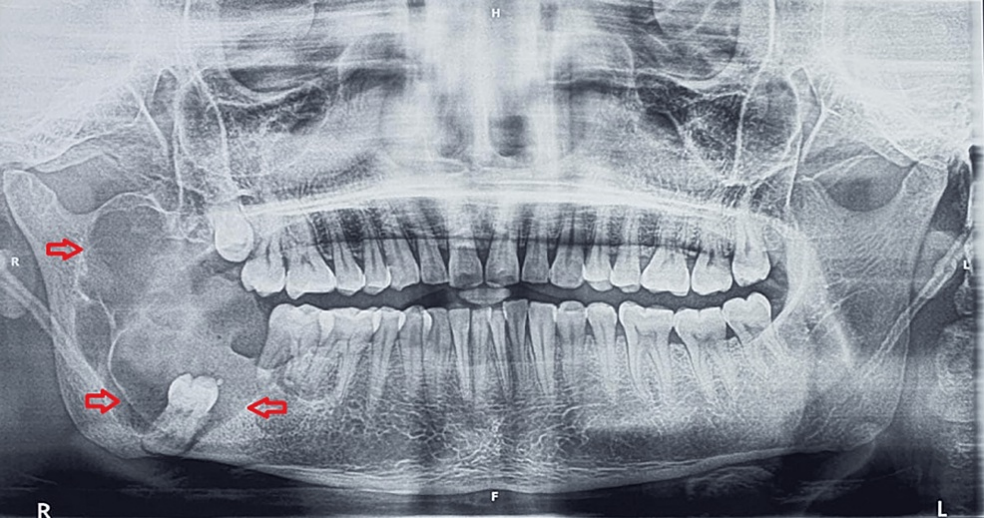
Multilocular radiolucency in right mandibular posterior region

An orthopantomogram (OPG) presented a multilocular radiolucency over the right body of the mandible, extending anteroposteriorly from the distal surface of 47 to the ramus and superoinferiorly from the sigmoid notch to inferior border of the mandible. The radiolucency was surrounded by a well-defined and corticated margins with subsequent displacement of 48 to inferior border of the mandible (Figure 4).

Histopathologic Features

**Figure 5:**
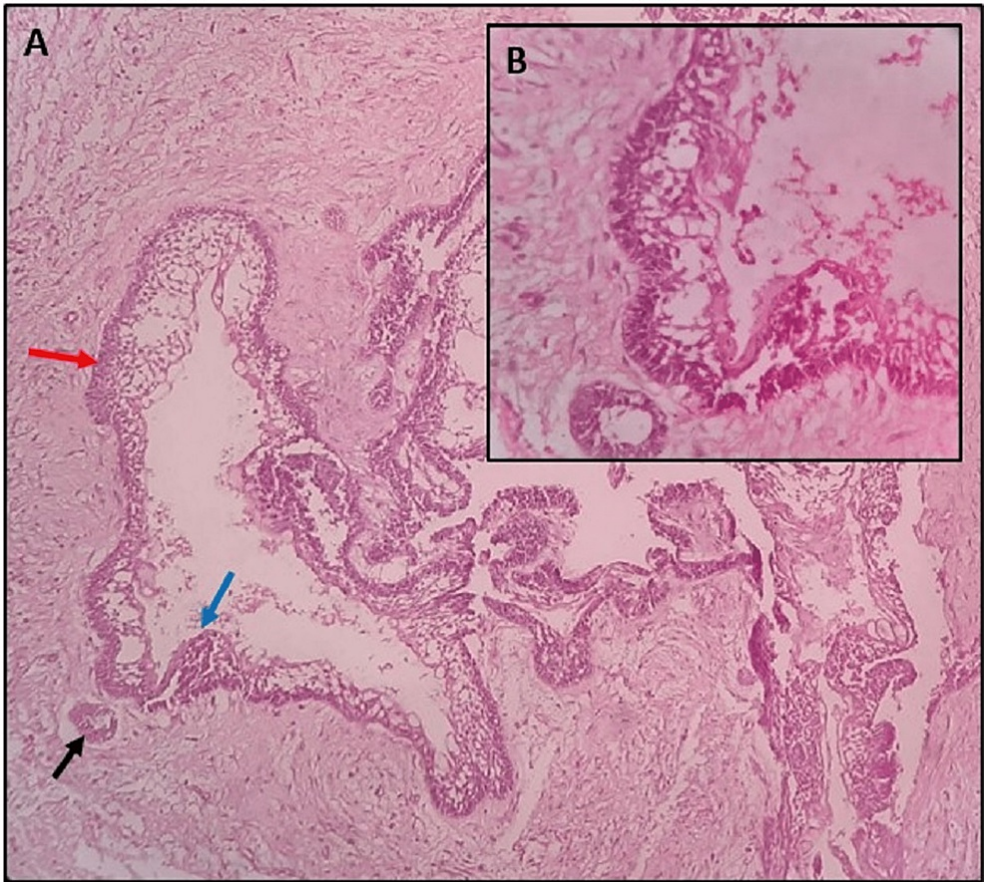
Luminal, intraluminal, and intramural variation. A: Features under low power view; B: High power view. Luminal, intraluminal, and intramural variations are marked by red, blue, and black arrows, respectively.

On histopathologic examination, Case 2 showed proliferation of tumor into luminal surface, cystic lumen, and connective tissue. Hematoxylin and Eosin (H&E) stained sections showed deeper connective tissue with few ameloblastic follicles showing typical peripheral tall columnar ameloblast‑like cells and central stellate reticulum like tissue. Histopathological features of present case were suggestive of UAs, Subtype 1.2.3 (Figure 5).

Treatment

The prognosis for UAs is good when it shows a cystic and well-localized pattern, surrounded by a fibrous capsule, and it is worst along with aggressive behavior when ameloblastomatous growth breaches the periphery of the fibrous capsule.The recurrence rate for UAs varies with different treatment modalities. The treatment protocol for UAs includes enucleation, curettage, marsupialization, and marginal resection. Considering the recurrence rate, radical resection was performed for the current case. After making a hockey stick incision, the sternocleidomastoid muscle, internal jugular vein, and accessory nerve were removed. Surgical defect was closed by the pectoralis major myocutaneous flap and reconstruction plates were used for load bearing fixation. Because of the long-term recurrence of UAs, postoperative follow up was also advised for a long duration.

Case 3

A 19-year-old patient reported to our institute with painful swelling over the mandibular right-back region for the past one-and-a-half years approximately. Pain was gradual at onset, dull, intermittent, and localized in nature. He was apparently alright one-and-a-half years back when he experienced swelling of the current size 5 x 3 cm approximately. Extraorally, facial asymmetry was present. The swelling extended anteroposteriorly from 1 cm behind the right commissure to the angle of the mandible and superiorly 1 cm below the ala-tragus line of the right side to the inferior border of the mandible of the same side. On inspection, it was roughly oval with diffused borders and tender on palpation. Intraorally, the lesion was extended from mesial of 44 to mesial of 46 with the involvement of the buccal sulcus. The overlying mucosa was inflamed with palpable right submandibular lymph nodes.

Radiographic Features

**Figure 6:**
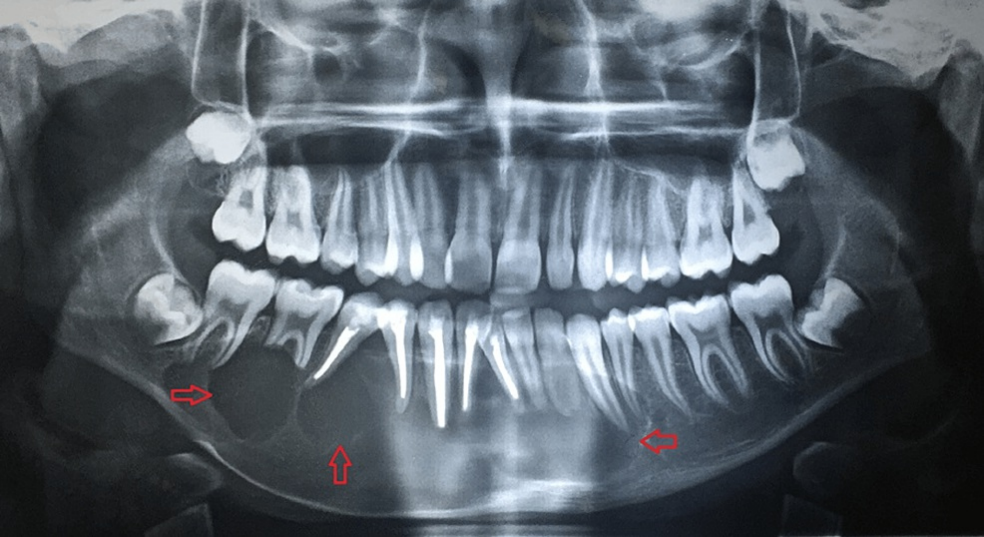
Multilocular radiolucency with soap bubble appearance (marked by red arrows)

An OPG displayed multilocular radiolucency depicting a soap bubble appearance over the left and right body of the mandible, extending in an anteroposterior direction from 34 to 47 and superiorinferiorly obliterating the root apices of 31, 32, 33, 34 and 41, 42, 43, 44, 45, 46 with thinning out the inferior cortex of the mandible (Figure 6).

Histopathologic Features

**Figure 7:**
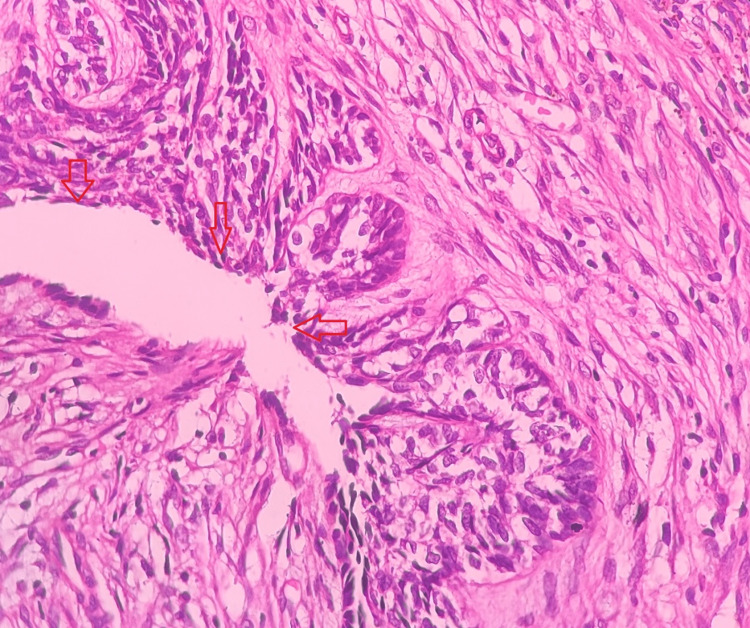
Intraluminal variation

On histopathology, Case 3 shows UAs, Subtype 1.2, luminal and intraluminal type of variation. H&E stained section showed an ameloblastomatous epithelium with a cystic lumen, in which the epithelial proliferations are seen.The intraluminal ameloblastomatous proliferation resembles a plexiform pattern (Figure 7).

Treatment

**Figure 8:**
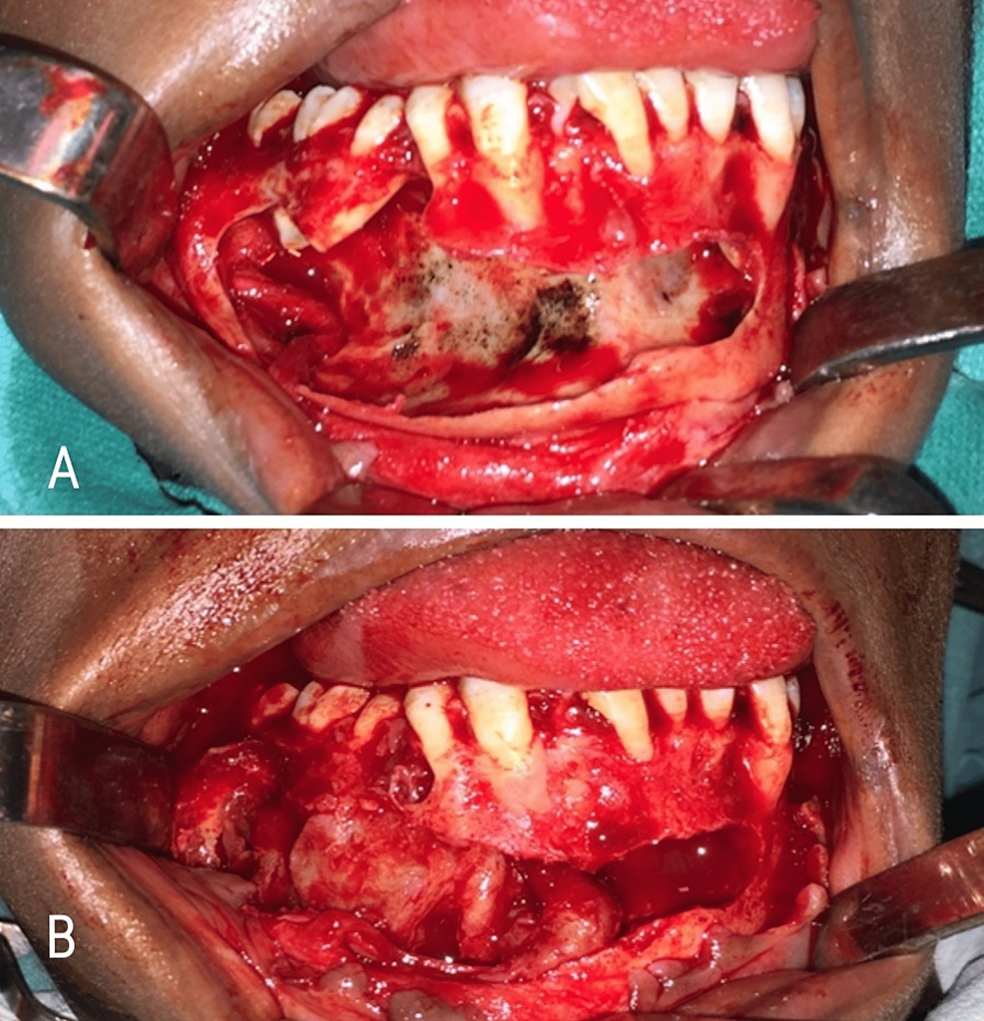
Enucleation with (A) curettage and (B) peripheral osteotomy

**Figure 9:**
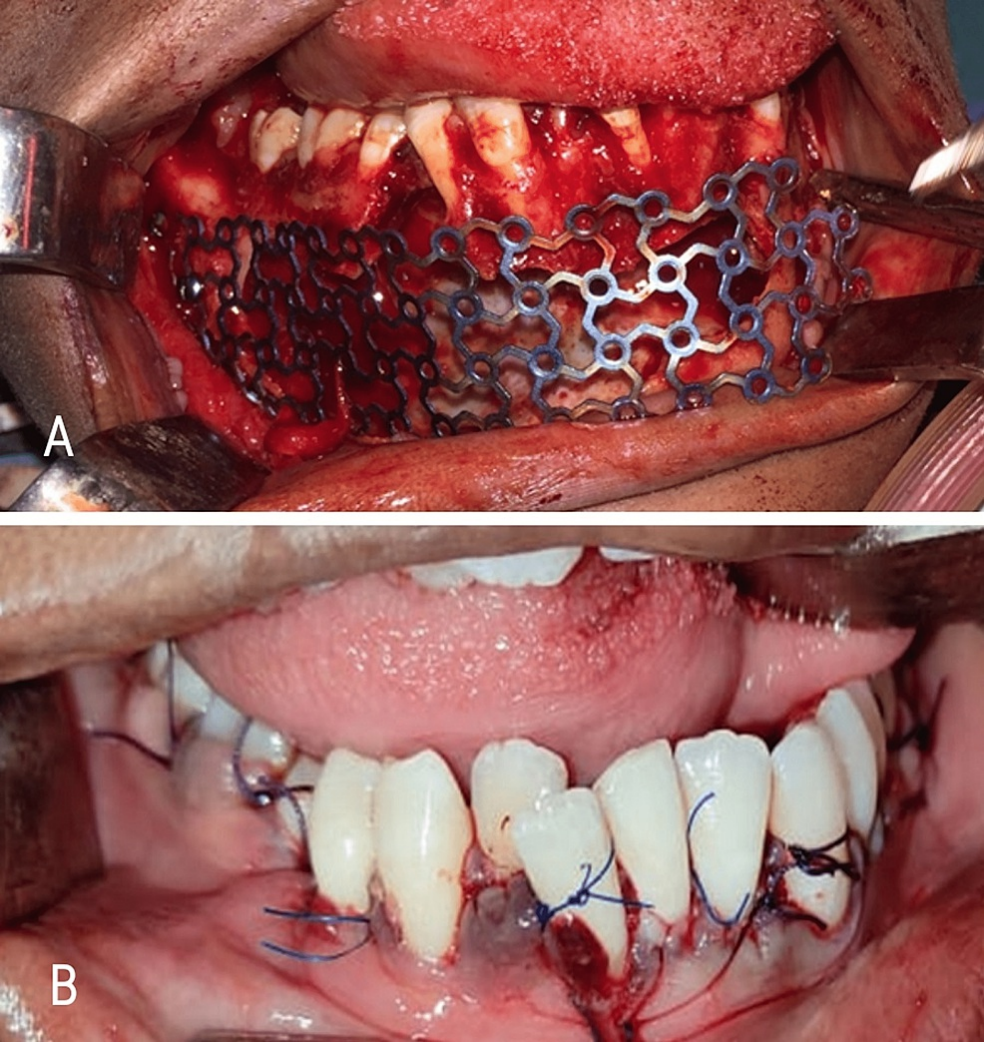
(A) Bone reconstruction with titanium mesh, and (B) Wound closure

The present case was managed by surgical enucleation with curettage (Figure 8A), and peripheral ostectomy (Figure 8B) of right mandibular body region. Retrograde filling was done with respect to 34-47 and root canal opening was done with 46. Reconstruction with titanium mesh (Figure 9A) was done along with beads of bone graft substitute (calcium sulphate dihydrate). Thorough irrigation with betadine and saline was followed by closure of the wound (Figure 9B).

Follow up 

**Figure 10:**
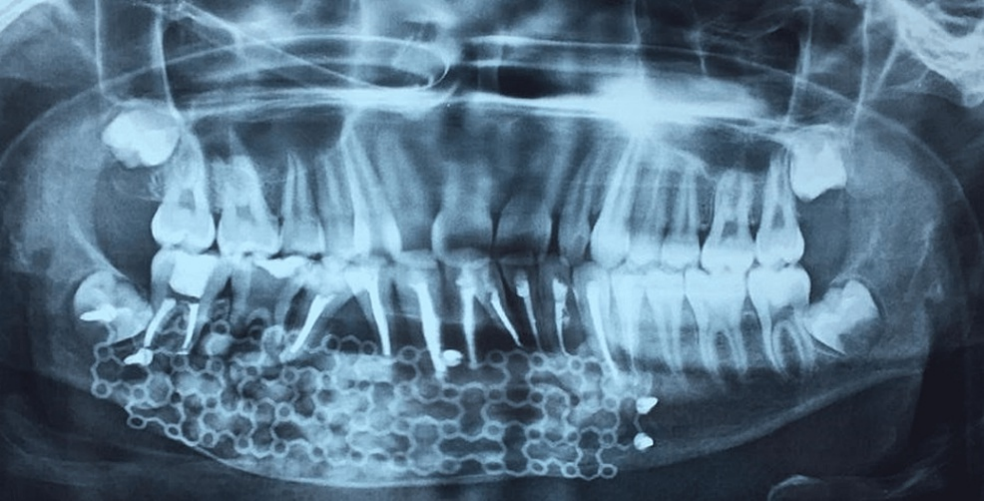
Postsurgical radiograph suggestive of bone formation

Postoperative radiograph showed radiopaque foci of bone generation with 44, 45 and mandibular anterior region was suggestive of bone formation (Figure 10). Due to surgical removal, the size of the lesion was reduced. The patient was discharged with prophylactic drugs, such as antibiotics, analgesics, antacids, and nutritional supplements, after all other parameters, including postoperative intraoral swelling, mouth opening, approximation of sutures, and bleeding, had been evaluated.

## Discussion

Ameloblastomas are locally aggressive, rare, and benign epithelial odontogenic tumors that form the category of epithelial tumors, including cystic as well as solid components with varied clinical and histopathological patterns. In 2017, the WHO classified ameloblastoma into unicystic and extraosseous/peripheral types. Our Case 1 ( Subtype 1.3) is in accordance with the case put forth by Mahalingam et al. (2020), which was a case of a mural variant of UA in a 32-year-old female patient with added emphasis on its histopathological importance [4]. Histopathological examination revealed a cystic cavity with ameloblastomatous epithelium and the connective tissue wall showed loose-to-densely arranged collagen fibers and mural islands. The presented case was managed by an aggressive mode of treatment same as conventional ameloblastoma. The authors had suggested that appropriate diagnosis of such cases should be done to minimize the high recurrence rate. Our Case 2 (Subtype 1.2.3) conforms with the case of Gupta et al. (2011) [1]. They illustrated a case of UA of the mandible in an 18-year-old female patient, managed successfully by right hemimandibulectomy and microvascular reconstruction with a free fibular flap. They put forth a question regarding the origin of UAs: whether it develops de novo or arises in an existing cyst. They proposed three pathogenic mechanisms for the evolution of UA arising from reduced enamel epithelium, from the dentigerous cyst, and due to cystic degeneration of solid ameloblastoma. They have further emphasized that to avoid misdiagnosis of UAs and to determine the origin, repeat and deeper biopsies should be advised to reveal the underlying tumorous proliferation as seen in the present case [1,4]. Histologically, peripheral ameloblastoma is similar to intraosseous ameloblastoma occurring in the odontogenic areas of jaws [5]. Our Case 3 (Subtype 1.2) is in accordance with the case illustrated by Nadendla (2012) [6]. He has presented a case of intraluminal and mural variant of UA. In the same case, a 35-year-old female reported swelling in the mandibular posterior region. Microscopic examination showed ameloblastomatous epithelium with a proliferation of epithelial cells into a cystic lumen. The lesion was managed by surgical excision followed by bone grafting. Further, he has concluded that as ameloblastoma presents multiple histological variants proper diagnosis with radiological, histopathological, and immunohistochemical studies should be considered to know the nature of the lesion and also differentiate the same from other cysts of odontogenic origin [6]. The most common variant of ameloblastoma is the unicystic type accounting for about 10-15% of all ameloblastomas. The pathophysiology of ameloblastoma is uncertain [7]. UAs are documented to originate either from reduced enamel epithelium of developing tooth or odontogenic cysts like dentigerous cysts. UAs can also arise from solid ameloblastomas undergoing cystic degeneration that forms multiple cysts which fuse to form single unicystic lesion [8]. Another classification proposed by Philipsen and Reichart is as follows: Subgroup 1, luminal UA; Subgroup 1.2, luminal and intraluminal; Subgroup 1.2.3, luminal, intraluminal, and intramural; and Subgroup 1.3, luminal and intramural [9]. All these four subgroups are seen in dentigerous as well as non-dentigerous variants. Local invasiveness plays a significant role in the high recurrence rate of ameloblastoma. To understand the biological behavior of UAs and to decide on various treatment modalities, we can take the help of various available biomarkers. Differentiating UAs from other cysts immunocytochemical markers for lectins and proliferating cells may be helpful [10]. To evaluate proliferative index, angiogenesis, protease activity, and behavior of UAs, various immunomarkers like Ki-67, CD34, matrix metalloproteinase-2, and matrix metalloproteinase-9 were used, suggesting high proliferative index in the mural ameloblastoma as compared to luminal and the intraluminal ameloblastoma [11]. Locally invasive and aggressive behavior of ameloblastomas is correlated to its tumor angiogenesis, proliferative capacity, neovascularization, and invasiveness properties [12]. B-Raf (BRAF) V600E is slightly more common in UAs (94%) than in solid ameloblastoma (74%) and fibroblast growth factor receptor 2 (FGFR2) mutations are found in ameloblastoma but not in UAs [13]. Immunomarkers are helpful to predict aggressiveness, which further helps to determine the diagnosis protocol and prognosis of the patient. UAs should be differentiated from calcifying epithelial odontogenic tumor, odontogenic keratocyst, central giant cell granuloma, and odontogenic myxoma in view of their prognostic behavior [9,14].

The clinical and radiological features of UAs are relatively characteristic. However, the diagnosis cannot be based on clinical and radiographic features alone. UAs clinically and radiographically resemble other odontogenic lesions but vary in histopathological pattern. Thus, histopathological examination is mandatory for arriving at a final diagnosis of UA. The histopathological classification of UAs is based on luminal, intraluminal, and mural proliferation of odontogenic epithelium. In our case study, the clinical presentation of all three UAs shows predilection to mandibular premolar to molar area. UAs usually show radiographic presentation as unilocular radiolucency. However, we observed multilocular presentation in all three cases of UAs. Histopathologically, we observed luminal and intramural as well as intraluminal proliferation in all our cases. Thus, a histopathological examination of UAs is very important for its treatment planning and predilection of prognosis. A thorough examination of complete tissue is mandatory to determine the proper diagnosis of UAs. The pathologist should examine the tissue sections carefully in an attempt to determine whether ameloblastoma has penetrated the wall of the cyst or not, so that the complications can be minimized. Long-term postoperative follow up is mandatory because the recurrence of UA may be long delayed. Especially post surgery, cases of mural UA should be reviewed periodically for recurrences. In context to surgical protocol, the luminal and intraluminal types can be treated by simple enucleation, but the mural types require special attention and have to be treated aggressively similar to conventional ameloblastomas. Further immunohistochemical studies help us to know the nature of the lesion and also to differentiate the same from other cysts of odontogenic origin. Apart from this, it is essential that studies should be conducted on a large scale in order to know the origin and nature of the lesion.

## Conclusions

The clinical and radiological features of UAs are relatively characteristic. However, there is variation in the histopathological features. UAs clinically and radiographically resemble other odontogenic lesions but vary in histopathological pattern. The histopathological classification of UAs is based on luminal, intraluminal, and mural proliferation of odontogenic epithelium. Clinical presentation of all three UAs shows predilection to mandibular premolar to molar. UAs usually show radiographic presentation as unilocular radiolucency. However, we observed multilocular presentation in all three cases of UAs. Histopathologically, we observed luminal, intramural, and intraluminal proliferation in all our cases. Thus, a histopathological examination of UAs is very important for its treatment planning and predilection of prognosis. A thorough examination of complete tissue is mandatory to determine the proper diagnosis of UAs.
